# Effects of Thermal Cycles on Interfacial Pressure in MV Cable Joints †

**DOI:** 10.3390/s20010169

**Published:** 2019-12-27

**Authors:** Raffaella Di Sante, Abbas Ghaderi, Alessandro Mingotti, Lorenzo Peretto, Roberto Tinarelli

**Affiliations:** Department of Electrical, Electronic and Information Engineering, Guglielmo Marconi Alma Mater Studiorum, University of Bologna, Viale del Risorgimento 2, 40136 Bologna, Italy; raffaella.disante@unibo.it (R.D.S.); abbas.ghaderi2@unibo.it (A.G.); lorenzo.peretto@unibo.it (L.P.); roberto.tinarelli3@unibo.it (R.T.)

**Keywords:** medium voltage, cable joint, temperature, interfacial pressure, XLPE, silicon rubber, thermal cycles, ageing

## Abstract

The use of medium voltage cable joints is mandatory when dealing with power cable faults and the installation of new lines. However, such an accessory is among the top causes of faults among the grid. To this purpose, one of the quantities monitored to understand the causes of such faults is the interfacial pressure between the insulating layers of the cable joint. In this work, the interfacial pressure between Cross-linked polyethylene (XLPE) and silicon rubber has been evaluated when the cable joint experiences thermal cycles. From the results, the pressure variation caused by the thermal cycles is demonstrated. Such a phenomenon may be connected to the generation of voids and weak spots that accelerate cable joint ageing. Therefore, proper comments and conclusions are drawn.

## 1. Introduction

Medium voltage (MV) cable joints have long been investigated due to the key role they play in underground power lines [1,2,3]. Their careful design and installation are critical to ensuring good electrical power transmission in cable systems, effective repair and installation of new lines. Being the weakest links in power lines, MV cable joints are responsible for the majority of faults [4], causing detrimental consequences for customers and distributed system operators (DSOs) or utilities.

In order to reduce the occurrence of failures in MV cable joints, it is of major importance to understand the mechanisms leading to loss of insulation. This can be obtained by specific measurement campaigns aimed at investigating the causes of fault, as is typically done for all electrical assets (e.g., instrument transformers [5,6,7,8], overhead lines [9,10], insulators [11,12,13], capacitors, [14] etc.). Good insulation depends on several factors, such as, for a certain voltage level, interface pressure and surface smoothness, materials, manufacturing, and installation. A given type of joint must exhibit good thermal performance [15,16,17] since this capability is related to cable ampacity. Different sensing systems have been proposed for continuous temperature monitoring in cable joints [18,19,20,21] and to be able to plan predictive maintenance.

Whilst temperature has been recognized as a major cause of cable joint failure [15,22,23], more recently, other parameters have been investigated, namely the tangent delta (Tanδ), interfacial pressure, and partial discharge (PD) level. In [24], an experimental setup is proposed for the estimation of tangent delta at ambient temperature. The same setup is then used to perform measurements in the 5–60 °C temperature range [25]. The experiments demonstrated a relationship between the temperature and the tangent delta, affecting the cable joint service life. In [26,27], different Tanδ extraction methods are presented.

The pressure between the insulating elements and the cable joint is also believed to affect the insulation effectiveness provided by the cable joint. The selection of materials plays a relevant role in this respect [28]. A method is available in literature to calculate and simulate the surface pressure [29]. The correlation between the interfacial pressure and tangent delta has been explored in [30]. The variation of the interfacial pressure with the ambient temperature was also investigated [31].

As for the PD, they are considered by several experts to be an effective method to assess the “healthy” status of the cable joints. Therefore, literature provides a high number of studies on this topic. For example, new PD extraction and analysis methods are presented in [32,33], while an on-line measurement setup is described in [34]. Based on these works [30,31], a correlation between the three physical parameters involved can be established.

In light of the above mentioned, the aim of this paper is to measure and evaluate the interfacial pressure between XLPE and SR when subject to thermal cycles. Such cycles aim at reproducing the ambient temperature variations that a cable joint suffers during its working life. From the results, compared also with the current literature, it can be appreciated a typical behavior, due in this case by the thermal cycles, that is cause of ageing acceleration of cable joints (as detailed below). 

The paper is structured as follows. In Section 2 the measurement setup and the details of its elements are described. The experimental tests are described in Section 3, while Section 4 is dedicated to the results analysis and comparison with consistent literature. In Section 5, comments on the obtained results and conclusion are drawn.

## 2. Measurement Setup

### 2.1. Overview

The measurement setup adopted in this work is the one developed and characterized in [31]. The main elements of the setup are the thermostatic chamber, capable of varying the temperature in the range 5 to 60 °C, and the cable joint under test (CUT). The CUTs are described in detail in next Section 2.2. The setup is completed with: (i) A Chauvin Arnoux CA863 contact thermometer to assess the temperature of the CUT and of the thermostatic chamber. The CA863 is a two channels device with operating temperature in the −50–1300 °C range, and it features a resolution and an accuracy of 0.1 °C and ±0.3% of the reading, respectively. (ii) A 24-bit National Instrument NI9239 Data AcQuisition board (DAQ) featuring an input range of ±10 V, a gain error and offset error of 0.03% and 0.008%, respectively. (iii) The sensing part of the setup includes 4 pressure sensors Flexi Force Standard Model HT201 characterized in [31]. Their main characteristics are: thickness of 0.203 mm, linearity up to 3 MPa, temperature drift of 0.088%/°C, and a non-linearity error of 3% of the full scale. A brief review of the typical pressure sensors and the reason for the choice made by the authors is provided in Section 2.3.

**Figure 1:**
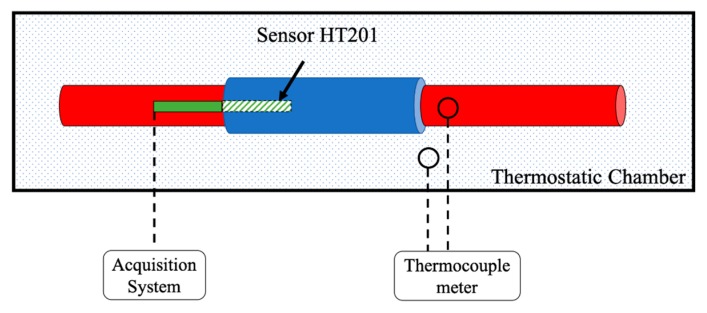
Simple schematic of the adopted measurement setup.

A simple schematic of the measurement setup is depicted in Figure 1.

### 2.2. Cable Joints

For the experimental tests, two cable joints built by two different manufacturers have been used. From here on now they will be referred as A and B for privacy reasons (and their sensors with $A_{1}$ and $A_{2}$, $B_{1}$ and $B_{2}$). The cable joints under test are both cold-shrinkable joints for MV cables. They have been installed, in laboratory environment, on two pieces of 130 mm^2^ MV, XLPE insulated cables. The CUT is composed of:

1. Metallic connector holding the two cable conductors.
2. Silicon Rubber (SR) as main insulating material covering the metallic connector and the XLPE insulating part of the cable. This silicon layer is covered by semi-conductive material.
3. A metallic meshed shielding covering the silicon rubber, which is used to maintain the ground electrical connection between the two portions of jointed cables.
4. The external cold-shrinkable layer.

**Figure 2:**
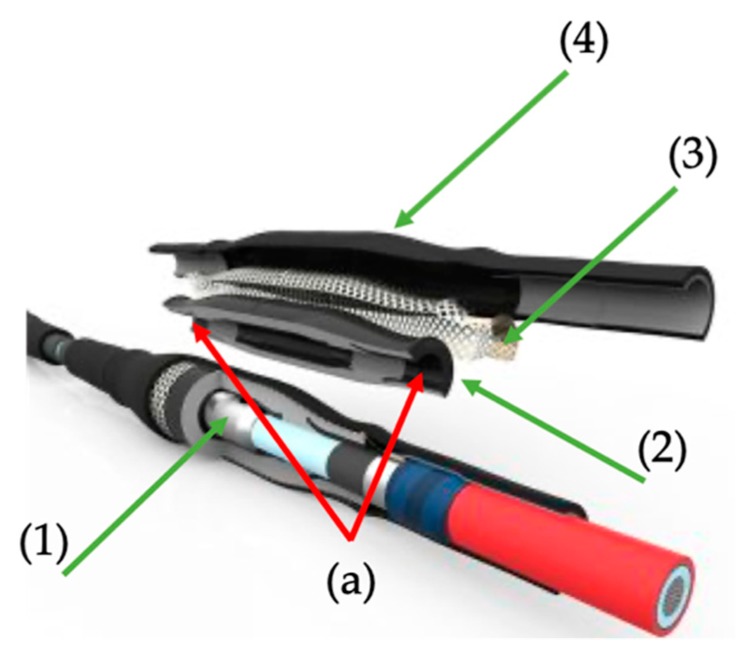
Cable joint structure with particular attention to the parts of interest.

The detailed cable joint parts are highlighted in Figure 2.

A few important comments can be made looking at Figure 2. Firstly, underneath the silicon rubber (Figure 2) a semi-conductive layer is placed on the metallic conductor to obtain a homogeneous electric field in that area. Secondly, at the two ends of the joint, a stress control layer, typically made of mastics, is placed over the XLPE insulation of the cable to modify gradually the electric field distribution (see Figure 2, element (a)).

In the CUT, the metallic meshed shielding has not been connected to the portion of cables shielding to allow the pressure sensors installation. However, neither voltages nor current have been applied for the following tests. Hence, the structure of the CUT has not been altered. Concluding, both cable joints A and B have the same structure of Figure 2. However, slight internal structural differences are possible due to the manufacturers’ expertise and methodology on the cable joint realization.

### 2.3. Pressure Sensors

Several types of pressure sensors are used in academia and industry for a variety of purposes. Such sensors are typically classified according to two criteria: the pressure measurement type, and the sensing principle implemented in the sensor.

Starting from the measurement type, three main categories can be described:

- Absolute measurement. This means that the reference point is the vacuum, and therefore, one side of the sensor itself is dedicated to it, while the other side is faced to the mean to be measured.
- Gauge measurement. This type of sensor shares the structure with the previous one, but it uses as reference point the atmosphere. Hence, when using this kind of sensor, the operator should always check that the air can flow inside the side of the sensor dedicated to the atmosphere.
- Differential measurement. According to their name, these sensors measure the pressure between two arbitrary points.

From the description, it is clear how the types of sensors share the basic principles and then each of the three has been developed to answer specific requirements of measurement.

As for the sensing principle, the most used technologies are the resistive, the capacitive, the optical, and the piezoelectric. The resistive and the capacitive type have the same working principle underlying the operation of sensor. In fact, the pressure induces a change of resistance/capacitance. The piezoelectric exploits the generation of charge to detect the pressure change in the sensor. Finally, the interferometry principle is used in optical sensor to appreciate pressure variations when the fiber optic is stressed.

**Figure 3:**
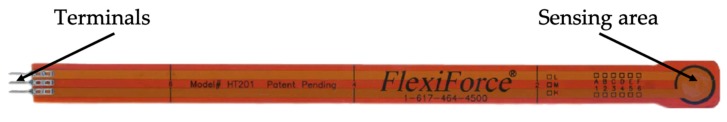
Picture of the adopted pressure sensor.

**Figure 4:**
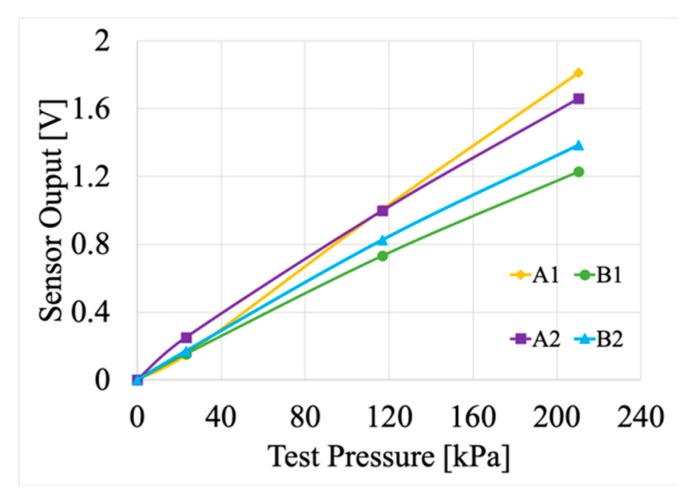
Characterization curves of the four sensors implemented inside the cable joint.

In light of the different available technologies, authors selected an enhanced piezo resistive force sensor adaptable to extreme temperatures, in the range −40 °C to 204 °C, and capable of linear measurements of pressures up to 3 MPa. The sensor, that provides a voltage proportional to the pressure, is depicted in Figure 3. In addition, the adopted technology allows to appreciate little variations of pressure (in a wide range of pressures), which is a key-feature for the cable joint monitoring. One possible disadvantage of the piezo resistive sensor is the fact that they could be temperature dependent. However, the characterization performed in [31] and the manufacturer specifications allowed to include the output variation due to temperature. For a full comprehension of the sensors’ behaviour, their characterization curves have been extrapolated from [31] and depicted in Figure 4. It represents the relationship between the voltage output and the applied pressure.

The last consideration is about the geometry of the system. The Flexi Force sensor has a thickness of 0.203 mm which makes it suitable for installation inside the CUTs without significantly changing their geometry, thus without interfering with the effect of temperature on the cable joints.

## 3. Description of the Experimental Tests

The aim of this work, as introduced in Section 1, is to assess the effect of thermal cycles on the pressure at the interfacial surface between XLPE and SR of MV cable joints. The thermal cycles aim at replicating the ambient temperatures variations affecting the cable joints, Therefore, in what follows, no current tests have been performed to avoid the superimposition of multiple thermal effects (other than the ambient temperature one) or any other effect due to the current. To this purpose, the two CUTs have been tested as follows. For 12 days the pressure measured by the four sensors has been daily collected at two temperature: room temperature (24 °C) and at 60 °C. This last temperature has been kept for 4 h to reach the thermal stability of the cable joint. The choice of repeating the tests for such a long period is aimed at obtaining a complete understanding of the thermal cycles’ effects on the cable joint and to avoid any anomalous measurements due to test performed just one single time. As for the temperature used for the tests, it has been fixed according to the typical working temperature of MV power cables, which is around 50 °C [35]. Furthermore, in [21], a measurement campaign for monitoring the temperature on the surface of underground MV power cables has been conducted. During such a campaign, performed in the south of Italy during the summer, the maximum temperature experience by the cable (and the cable joint) was 40 °C.

After the collection of the measurements, the CUTs were left to cool naturally at 24 °C until the next-day set of measurements. To ensure the thermal stability of the CUT, the measurements were performed only when the thermocouple gauging the air temperature and the one inserted in the joint have the same value (after 4 h). For all tests, 250 measurements of pressure were acquired from each sensor installed in the CUT. In addition, pressure has been collected, for both CUTs, at the same time to guarantee the same temperature conditions in all tests.

This represents the first part of the experimental measurements on the CUTs. After 12 days, the CUTs were kept at room temperature for 43 days without performing any pressure test. Then, another measurement at room temperature has been performed to understand the behavior of the CUTs. Such a measurement has been repeated again after 31 and 49 days, always at an ambient temperature of 24 °C.

**Figure 5:**
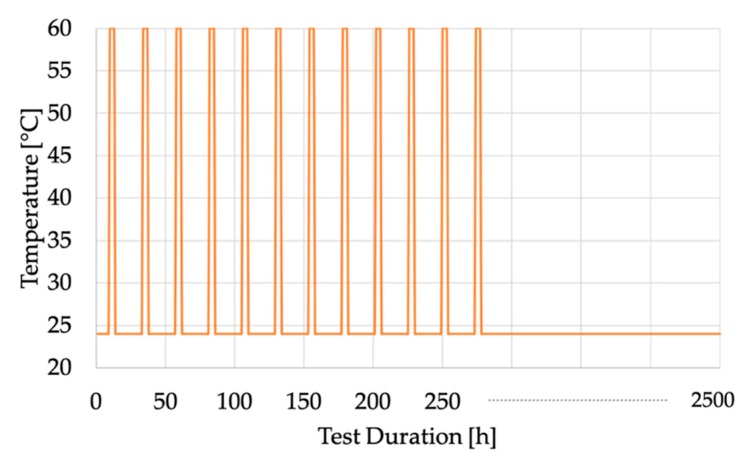
Temperature vs. duration of the tests on the CUTs.

To better clarify the overall experimental procedure, in Figure 5 the temperatures set during the tests have been plotted together with the time window of the entire measurement campaign. In the graph, the second part of the tests, which is at 24 °C without changes, has been compressed for the sake of clarity.

Overall, the experimental campaign on the CUTs lasted more than three months. Such a duration has been chosen to assess the interfacial pressure behavior of the CUTs when subjected to thermal cycles. It is worth to highlight that, for the duration of the tests, the CUTs have not been moved at all, to replicate the actual condition of the grid. In fact, MV cables, buried underground, are not subjected to any displacement in their life cycle (with the exception of a fault happening on the portion of cable considered).

## 4. Results & Discussion

### 4.1. Measurement Results

The pressure measurements collected in the three-month period are listed in Table 1. It contains on the first column the list of days when the measurements have been performed and, for each temperature, the mean value of the acquired pressures (250 each). As mentioned in Section 2.1, each CUT mounts two pressure sensors. These have been distinguished as $A_{1}$ and $A_{2}$ for the CUT *A*, and $B_{1}$ and $B_{2}$ for the CUT *B*.

The pressure measurement results are accurate for the purpose of the work. In fact, the overall uncertainty affecting the pressure measurement is 1 kPa, and therefore predominant compared to the calculated standard deviation of the mean, which has been neglected and not reported here (between 0.02 and 0.04 kPa). This last range of values for the standard deviation has been confirmed for the entire duration of the test; therefore, the stability and repeatability over the time is ensured.

From Table 1, it is worth highlighting two interesting comments: one about the two CUTs, and one about the two sensors of a single CUT. First, comparing the CUTs, a difference between the measured pressures is evident. Such a behavior was already found in [31] and it is due to the different manufacturers’ ways of preparing the cable joints, considering that the material adopted by the two manufacturers is the same (with the exception of possible chemical variation due to the manufacturers expertise). Obviously, the manufacturers do not provide any details on the interfacial pressure of their joints; hence, the only source for such an information is from a measurement campaign, as it has been done in this work, or from existing literature [36]. However, as for the reliability of the presented pressure measurement, the two cable joints have been manufactured by experts that build and repair joints in the field.

**Figure 6:**
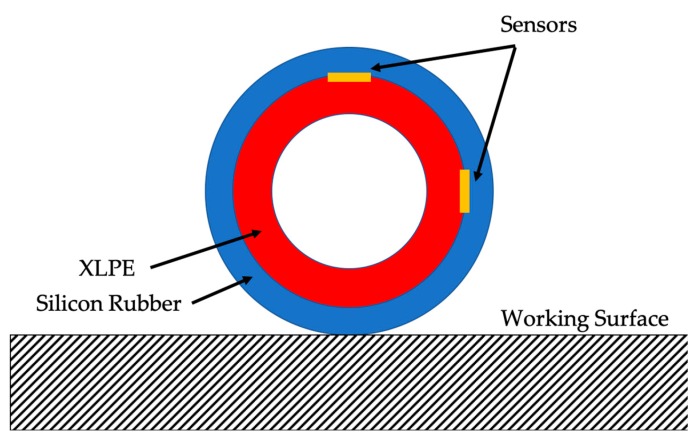
Positioning of the two sensors inside the CUT and of the CUT itself.

The second comment concerns the two sensors inside the single CUT. In fact, slightly different values have been collected in both CUTs. This effect is reasonable considering the 90° angle between two sensors of the same CUT (see Figure 6). As a matter of fact, the gravity and the positioning of the CUT over the working surface are affecting the measured pressure.

However, in this specific work, attention is not given to the absolute value of the pressure but on its variation vs. temperature.

**Figure 7:**
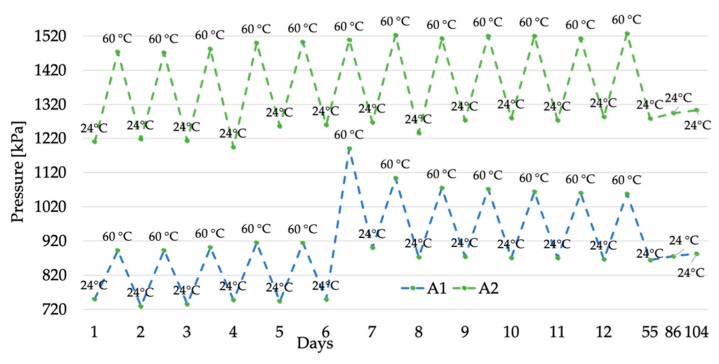
Pressure measurement results for CUT *A*, sensors $A_{1}$ and $A_{2}$.

**Figure 8:**
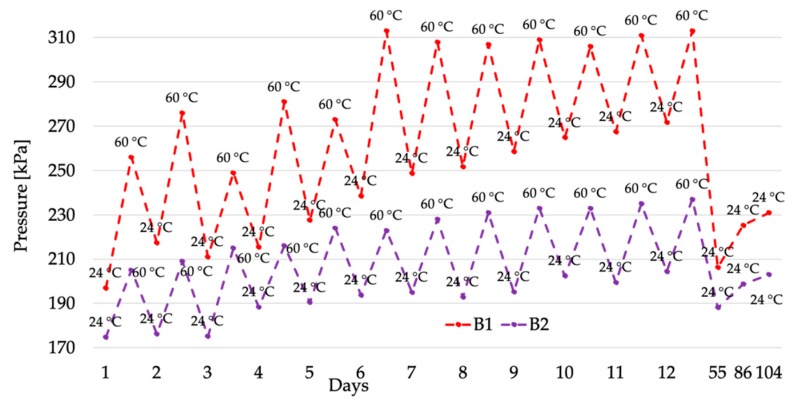
Pressure measurement results for CUT *B*, sensors $B_{1}$ and $B_{2}$.

To increase the readability of the results and to provide further comments on them, the pressure measurements have been plotted in Figure 7 and Figure 8 for the CUT *A* and *B*, respectively.

After the specific comments on the absolute value of the results, it is important now to highlight the trend of the pressure in the two CUTs. A general statement is that the thermal cycles have a non-negligible effect on the interfacial pressure of the cable joints. In particular, all four sensors, hence both joints, experience an increase of the pressure as the number of cycles increase. If this phenomenon is clear for sensors $A_{1}$, $B_{1}$, and $B_{2}$, from the graph it is less appreciable for sensor $A_{2}$. To this purpose, the percentage variation between day 1 and day 12 has been calculated for the four sensors. The results are 15.5, 6.0, 38.0, and 16.9% for $A_{1}$, $A_{2}$ $B_{1}$, and $B_{2}$, respectively.

Overall, the first 12-days temperature cycles stressed the CUTs causing an interfacial pressure variation up to 40%. Such a pressure variation is critical when looking at the second-phase test results. In fact, after 43 days the room-temperature measured pressure dropped significantly compared to the one collected in the 12-day period. However, the detected drop is not sufficient to restore the interfacial pressure of the joint to the values obtained before the thermal cycles. In detail, percentage variations with respect of the first pressure measured at room temperature are 15.2, 5.6, 4.7, and 7.6%, for $A_{1}$, $A_{2}$ $B_{1}$, and $B_{2}$, respectively.

The effects of the thermal cycles are persistent even after more than 80 days after their end. As it is evident from Figure 7 and Figure 8, the pressure remains quite stable in all tests; with the exception of CUT *B*, which exhibits a slight pressure increase. Therefore, it can be concluded that the combination of the thermal cycles and the memory effect of the materials adopted to build a cable joint cause a variation of the mechanical properties of the joint itself.

### 4.2. Discussion & Comparison

#### 4.2.1. Analysis of the Results

What has been obtained in Table 1 can be described starting from the expression of the expansion that the material is suffering when subjected to temperature variations:

(1) $$\Delta r_{x}=\alpha_{x}r_{0x}\Delta T_{x},$$

(2) $$\Delta r_{s}=\alpha_{s}r_{0s}\Delta T_{s}.$$

For the sake of simplicity, Equations (1) and (2) refer to the linear expansion, but the concept can be extended also for the volumetric expansion. In Equations (1) and (2), the subscript *x* and *s* refer to XLPE and SR, respectively. Such subscripts have been associated to the temperature variation experienced by the materials ΔT, the initial radius of both insulating materials $r_{0}$, the thermal expansion coefficient α, and finally the radius variation caused by the temperature Δr.

Considering that the pressure increases with the temperature, the terms in Equations (1) and (2) must be analyzed. Firstly, it can be assumed that $\Delta T_{x}\;=\;\Delta T_{s}\;=\;\Delta T$ due to the measurement process. In fact, the thermocouple has been placed at the interface between XLPE and SR, and the measurements have been performed only when the thermal stability is reached (as detailed above). Therefore, the contribution of the temperature to the thermal expansion of the insulation materials can be considered the same for both of them. Secondly, in terms of radius, XLPE and SR are laying one over the other; therefore, their layers subjected to the expansion can be considered equal.

Hence, it can be observed that the expansion in outer and inner direction (with respect to the cross section of the cable) has a similar contribution for both XLPE and SR. What differentiates Equations (1) and (2) is simply the thermal coefficient of the two materials. In light of these assumption, and dividing Equation (1) by Equation (2):

(3) $$\frac{\Delta r_{x}}{\Delta r_{s}}=\frac{\alpha_{x}}{\alpha_{s}},$$

the reasons of the increase of pressure in the interfacial surface of the joint can be explained. Typically, $\alpha_{x}$ and $\alpha_{s}$ vary in the 4–7 $\times 10^{-4}$ 1/°C range, depending on the particular materials and by their cross-linking level [37] adopted by each manufacturer. Therefore, the pressure variation measured in the test can provide different results if $\alpha_{x}\;⋚\;\alpha_{s}$ and from their absolute value. In particular, the different percentages provided in Section 4.1 can be associated to different values of α related to the manufacturers’ materials.

However, considering that manufacturers are typically reluctant to provide technical specifications of their products and of their manufacturing process—as in the case of the cable joint where the chemistry is also involved—what can be concluded is that the more similar the α coefficients are, the more the homogeneous pressure variation will be between the insulating layers of a cable joint. In conclusion, the ideal situation would be of having insulating materials with negligible difference between their α values.

As a final comment, the above discussion assumes that α is independent of the temperature. Such assumption can be considered relevant in this work due to the level of temperature used in the tests (60 °C), which is far lower thant the glass transition temperature of XLPE (typically around 130 °C) and SR (90 °C, provided by the manufacturers), hence far from the values at which α starts to experience the effect of temperature [38].

#### 4.2.2. Comparison with Literature

The obtained results clearly confirm those obtained in [31] in terms of relationship between temperature and pressure. Furthermore, the increase of pressure measured during the tests can be related to the outcomes of [30]. In such work, it has been demonstrated that a pressure increase reduces the Tanδ value in the cable joint. This is due to the fact that the increase of pressure in the cable joint results in the leakage current reduction among the joint layers, hence in a reduction of the Tanδ [30]. As a result, the dielectric properties of the joint, and therefore of its expected life and reliability, are changed and in particular they increase. Based on this work, further evidence emerges, allowing for a more comprehensive analysis. The cable joint exposure to thermal cycles and related pressure variation results into a higher level of the cable joint pressure at room temperature after the thermal cycles. The overall effect could be positive if assessed on the final absolute value of the pressure; in fact, higher pressure results in lower Tanδ [30]. However, the always varying pressure due to the thermal cycles is changing the mechanical structure of the cable joint, increasing the creation of voids and weak spots inside the insulating materials.

Quite different results have been obtained in a similar work [36]. The main discrepancies are: (i) The pressure sensor adopted is a load cell. Even if a miniature cell has been used, such a sensor changes the delicate internal structure of the cable joint, thus modifying the operating conditions and the original interfacial pressure. (ii) Thermal cycles have been performed varying the temperature between a minimum and a maximum, for three different maximum temperatures: 75 °C, 90 °C, and 130 °C. Such temperatures are really close to the glass transition and melting temperature of the adopted materials and therefore, unsuitable for tests aimed at replicating the actual conditions experienced by the cable joint (see [35]). (iii) The materials adopted in the early 2000 s are quite different from those manufactured today. Therefore, a simple comparison is not possible due to the technological improvements achieved in recent years.

**Figure 9:**
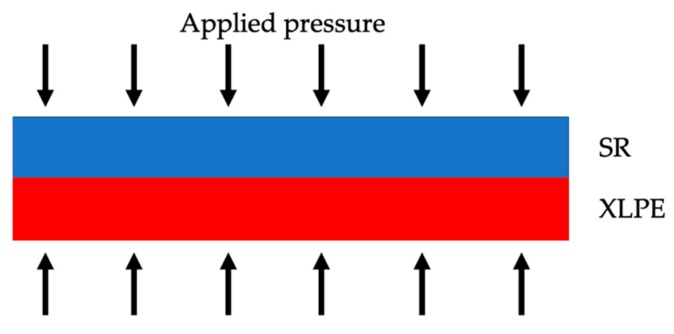
Simple schematic of how pressure has been tested on the XLPE/SR samples.

Another two interesting works on the interfacial pressure in MV cable joints, [39,40], demonstrated how the increase of pressure causes an augmented breakdown voltage in two samples of XLPE and SR, adjusted one over the other. This result confirms what obtained in [30], however, a straightforward conclusion cannot be drawn without testing the complete cable joint system. In fact, in [39] the pressure increase has been applied on parallelepiped samples, as shown in Figure 9, whilst [30] considers the pressure acting on the overall surface of the cable joint.

The obtained final effect is totally different: Whilst in [39,40] the pressure acts on the samples reducing the voids, thus increasing the breakdown voltage, in [30] the pressure acts on the overall cable joint, modifying the internal structure and confirming the positive effect of the pressure. However, frequent pressure change at the interfacial surface XLPE/SR is a negative effect from the dielectric point of view. In fact, the pressure variation contributes to the creation of voids and material deformations that can lead to the increase of partial discharges till the complete breakdown inside the insulating material.

**Figure 10:**
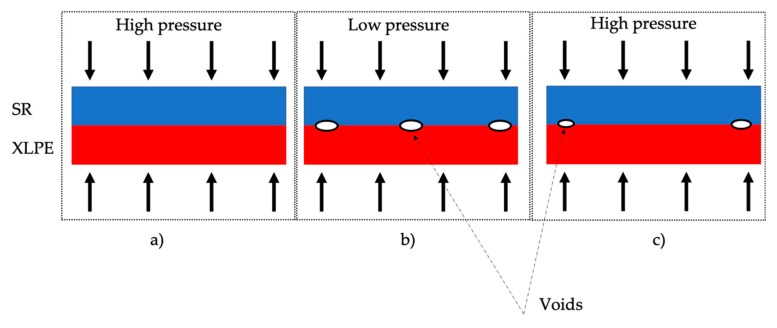
Simple schematic of void creation at the interface of XLPE and SR, subject to pressure variations. (**a**) First stage with high pressure, (**b**) Second stage with low pressure and (**c**) Third stage, again with high pressure.

To detail such a critical process, Figure 10 is of help. It shows the voids creation process at the XLPE/SR interface when the applied pressure is varying. The figure refers to a small cross-section of the insulating system of a cable joint; hence what showed in the picture can be extend to the entire cable joint.

Starting from Figure 10a, the initial condition (supposed high) presents no voids. Afterwards, when a temperature drop is measured, hence a consequent pressure drop, at the interface between the materials voids may appear (Figure 10b). As a matter of fact, the different thermal expansion coefficient of XLPE and SR cannot guarantee their perfect adherence. Then, if the pressure increases again, as shown in Figure 10c, the created voids may be reduced, but there is no certainty about their disappearance. Furthermore, during the presence of voids, the partial discharge phenomenon could have been already started and worsened the material conditions (even enlarging the voids).

In light of this, studying the cable joints is always a difficult task considering the complexity of their structure. In fact, several physical quantities affect their operation during their lifecycle and their influence has to be precisely evaluated trying to replicate at best the actual operating conditions.

## 5. Conclusions

The causes of fault in MV electrical assets, and in particular in cable joints, is a current topic tackled in literature. To this purpose, the authors addressed in this work one of the main influence quantities affecting the joints’ operational life, i.e., the temperature. For three months, the two cable joints under test have been subjected to thermal cycles, varying the ambient temperature. The obtained results, compared and discussed with the current literature, showed that there is a strong relationship between the interfacial pressure cable/cable joint and the ambient temperature. In particular, when subjected to thermal cycles, such an interface experiences an increase of pressure when the temperature increases. Vice versa, it relaxes when the temperature decreases. Consequently, such a phenomenon can be directly correlated to the creation of voids and weak spots in the cable joint insulation (as confirmed by the literature), due to the continuous pressure variation inside the joint. Such an effect increases the ageing of the cable joints, shortening their lifecycle and causing damage to the DSOs and their customers.

Finally, the obtained results present a first step for further studies that may correlate the effect of other influence quantities on the ageing of cable joints. Furthermore, in light of the results, the material selection and the manufacturing procedures of the cable joint producers may be improved to better match the physical phenomena involved in the operation of the cable joint. Accordingly, the conclusions herein contribute towards the objective of reducing the number of faults in medium voltage cable joints.

## Figures and Tables

**Table 1:** List of all pressure measurement results for the 2 CUTs.

Days	Pressure (kPa)							
	24 °C				60 °C			
	$A_{1}$	$A_{2}$	$B_{1}$	$B_{2}$	$A_{1}$	$A_{2}$	$B_{1}$	$B_{2}$
**1**	750	1210	196	174	892	1473	256	205
**2**	729	1218	217	176	893	1471	276	209
**3**	735	1213	210	175	901	1482	249	215
**4**	747	1194	215	188	915	1500	281	216
**5**	743	1256	227	190	914	1502	273	224
**6**	748	1260	238	193	1191	1508	313	223
**7**	900	1267	248	195	1104	1523	308	228
**8**	872	1236	251	192	1075	1512	307	231
**9**	874	1273	258	195	1072	1520	309	233
**10**	870	1279	264	202	1064	1520	306	233
**11**	870	1273	267	199	1060	1513	311	235
**12**	866	1283	271	204	1058	1528	313	237
**55**	864	1278	206	188	-	-	-	-
**86**	875	1294	225	198	-	-	-	-
**104**	883	1303	231	203	-	-	-	-
